# Symmetry-protected transport in a pseudospin-polarized waveguide

**DOI:** 10.1038/ncomms9183

**Published:** 2015-09-23

**Authors:** Wen-Jie Chen, Zhao-Qing Zhang, Jian-Wen Dong, C. T. Chan

**Affiliations:** 1 Department of Physics, Institute for Advanced Study, The Hong Kong University of Science and Technology, Hong Kong, China; 2 State Key Laboratory of Optoelectronic Materials and Technologies, School of Physics and Engineering, Sun Yat-Sen University, Guangzhou 510275, China

## Abstract

If a system possesses a spin or pseudospin, which is locked to the linear momentum, spin-polarized states can exhibit backscattering-immune transport if the scatterer does not flip the spin. Good examples of such systems include electronic and photonic topological insulators. For electromagnetic waves, such pseudospin states can be achieved in metamaterials with very special artificial symmetries; however, these bulk photonic topological insulators are usually difficult to fabricate. Here we propose a paradigm in which the pseudospin is enforced simply by imposing special boundary conditions inside a channel. The symmetry-protected pseudospin states are guided in air and no bulk material is required. We also show that the special boundary conditions can be implemented simply using an array of metallic conductors, resulting in spin-filtered waveguide with a simple structure and a broad working bandwidth. We generate several conceptual designs, and symmetry-protected pseudospin transport in the microwave regime is experimentally indicated.

## Abstract (web summary)

Designing robust electromagnetic channels where transport is unidirectional is still a challenge. Here, the authors propose a new paradigm for spin-filtered waveguides in air, avoiding the use of special bulk materials.

## Graphical abstract

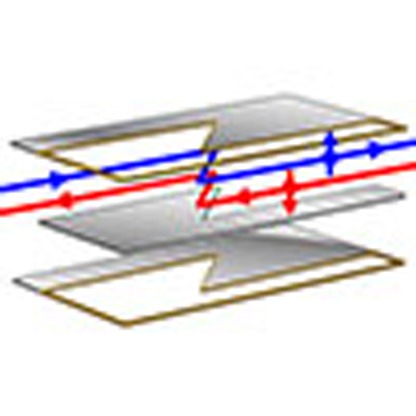

One-way transport of light has been actively pursued to suppress backscattering in optical devices. Most of the previously reported one-way waveguides were based on magneto-optic effects^1^,^2^,^3^. Disorder does not introduce backscattering in these nonreciprocal systems as backward propagating modes are absent because of the breaking of time-reversal symmetry. Nonreciprocities of electromagnetic (EM) waves on the basis of other mechanisms, including optical nonlinearity^4^,^5^,^6^ and indirect interband photonic transitions^7^,^8^,^9^, were also investigated, although they do not support scattering-free transport. One-way edge states of magnetic photonic crystals^10^,^11^,^12^,^13^, which are analogous to the quantum Hall effect^14^,^15^, have been predicted and observed. Robust transport can also be realized without breaking time-reversal symmetry^16^,^17^,^18^,^19^,^20^,^21^,^22^ in the form of ‘photonic topological insulators'^23^,^24^,^25^,^26^,^27^,^28^. Spin-filtered edge states at the boundary of such systems are protected by the nontrivial topology of bulk states. They are robust against backscattering, provided that scatterers preserve the pseudospin. All of these strategies require a special bulk material to guide light in a special way. The natural question is whether ‘pseudospin' transport is possible with light guided in air. In addition, spin-dependent coupling has been used to realize an all-optical photonic switch^29^ and directional spontaneous emission^30^ in the optical regime recently.

In this paper, we propose a spin-filtered waveguide without using any bulk material. Wave propagation in the waveguide is robust against deformations that do not induce spin flip. The waveguide possesses a simple structure and a broad working bandwidth.

## Results

### Concept of pseudospin-filtered waveguide

Consider a time-reversal invariant system whose relative permittivity and permeability satisfy a mirror reflection symmetry of 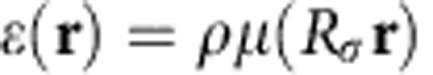. Here 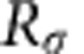 is a mirror operator and *ρ* is a global constant in the whole space. For the sake of simplicity, we assume that the mirror plane is the *xy* plane (for the conciseness of the equations) and *ρ*=1 (as we will discuss wave guided in air below), that is, *ɛ*(*z*)=*μ*(−*z*). The Maxwell equations then reduce to two decoupled equations:


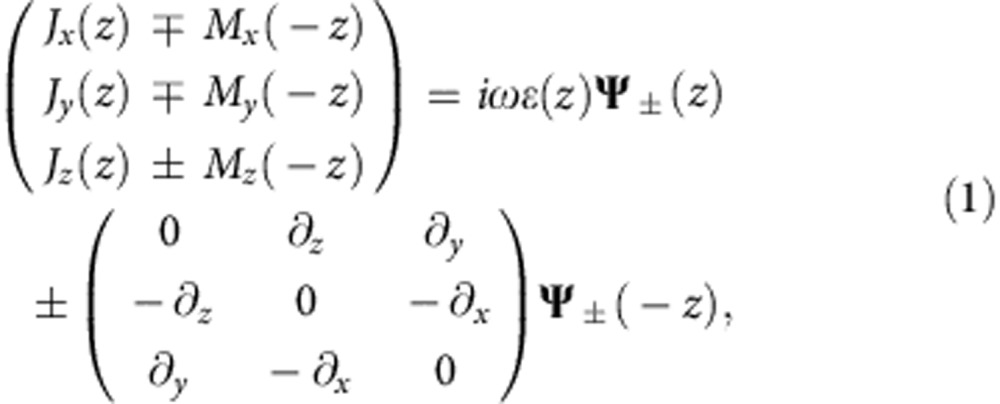


where


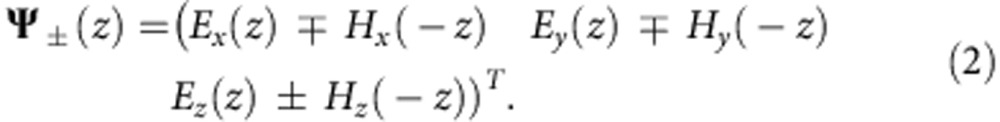


**Figure 1:**
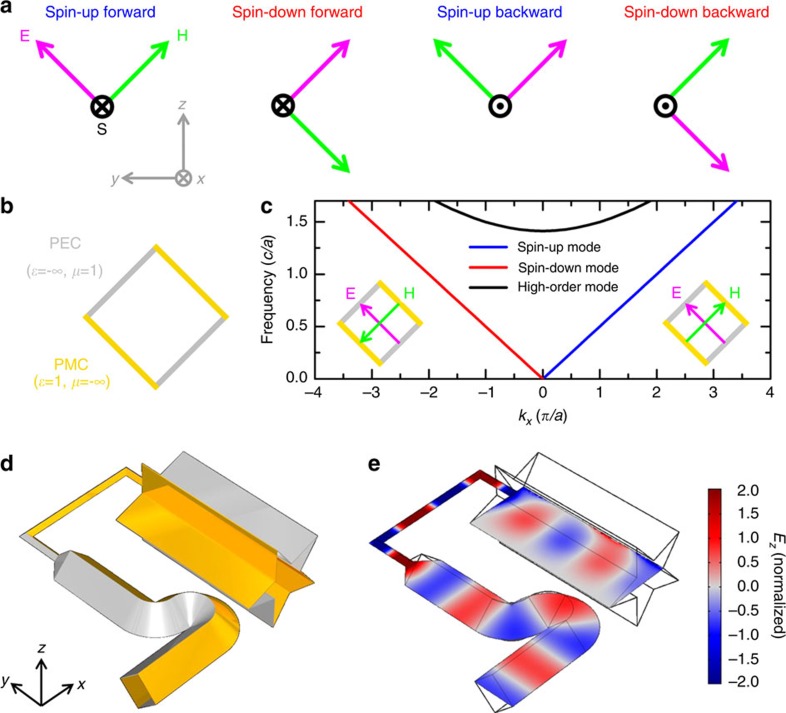
Concept of pseudospin-filtered waveguide. (**a**) Four eigenmodes propagating along the *x* direction in air. Air does not have the spin-filtered feature because it supports spin-up (spin-down) modes in both forward and backward directions. One can achieve a spin-filtered waveguide by applying boundary conditions. (**b**) Cross-sectional view of a square waveguide with PEC and PMC boundaries. The side length of the square cross-section is 
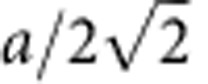
. (**c**) Waveguide dispersion. Only two TEM modes are allowed below the cutoff frequency of a spin-degenerate high-order waveguide mode 
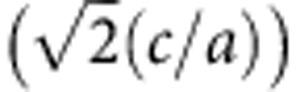
. *c* is the speed of light. The forward mode propagating along the +*x* direction is pseudospin-up polarized as its eigenfield (right inset) satisfies the relations of *E*_*x*,*y*_(*z*)=−*H*_*x*,*y*_(−*z*) and *E*_*z*_(*z*)=*H*_*z*_(−*z*). The backward mode is pseudospin-down polarized (see the eigenfield in left inset). The square waveguide is hence a spin-filtered channel. Its transport is robust against backscattering, unless the scatterer or deformation flips the pseudospin. (**d**) Geometry of a deformed waveguide. EM wave with 45°-tilted polarization enters the bottom of the deformed waveguide, which consists of a square waveguide successively connected to an S-shaped bend, another square waveguide with very small (1/25) cross-section and a star-shaped waveguide. As the *ɛ*(*x*, *y*, *z*)=*μ*(*x*, *y*, −*z*) symmetry is preserved despite these deformations, the spin-up mode and the spin-down mode are decoupled, and no backscattering can occur. (**e**) *E*_*z*_ field pattern in the deformed waveguide normalized by the incident field amplitude.

Variables *x* and *y* are omitted for conciseness. *J* and *M* denote electric and magnetic currents. For simplicity, *E*(*M*), *H* (*J*) and *ω* are normalized by 
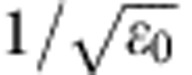
, 
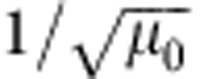
 and 
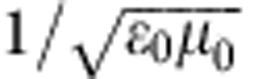
, respectively, so that electric and magnetic fields (currents) have the same dimension. **Ψ**_+_ and **Ψ**_−_ are referred to as pseudo spin-up and spin-down states, as they are linked by time-reversal symmetry and decoupled from each other. By the definition of spin-up (spin-down) state in equation (2), the *E*_*z*_ field component at point (*x*, *y*, *z*) and the *H*_*z*_ field component at point (*x*, *y*, −*z*) are in phase (out of phase), while the *E*_*x*,*y*_ field component and the *H*_*x*,*y*_ field component are out of phase (in phase) so that the electric field and magnetic field distributions of the spin-down (spin-up) state form a mirror (antimirror) reflection about the *xy* plane, that is, **H**=*σ*_*z*_**E** (**H**=−*σ*_*z*_**E**). Equation (1) describes the relations between source and field. The spin-up and spin-down states can be excited separately, depending on whether the electric current and the magnetic current are in phase or out of phase. Note that *ɛ*(*z*)=*ρμ*(−*z*), which ensures the decoupling of the pseudospin-up and spin-down subsystems, is a necessary albeit insufficient condition for spin-filtered transport. This is because the pseudospin is not necessarily locked to the momentum. For example, air satisfies the *ɛ*(*z*)=*μ*(−*z*) symmetry but it obviously is not a spin-filtered channel. Figure 1a plots the eigenmodes of an EM wave propagating along the *x* direction in air. There are four linearly polarized plane waves with two spin-up and two spin-down modes according to the definition of pseudospin in equation (2). Because the spin-up and spin-down waves can propagate in both the (+*x*) and (−*x*) directions, air does not have the spin-filtered feature.

To realize spin-filtered transport, we must design a channel that supports only the spin-up forward mode and the spin-down backward mode. This can be achieved by applying boundary conditions. Consider a square waveguide filled with air, as shown in Fig. 1b. The four walls are alternatively made up of perfect electric conductors (PECs) and perfect magnetic conductors (PMCs). The boundary condition only allows the spin-up forward and spin-down backward modes to exist, and hence the pseudospin is locked to the wave vector and the square waveguide becomes a spin-filtered channel. The PEC–PMC boundary conditions have been used in waveguides^31^,^32^ where the transport property relies on the electrically small *ɛ*-near-zero materials that fill the waveguide. Figure 1c depicts the dispersion relation of our square waveguide. The side length of the square cross-section is 
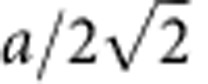
. Below the cutoff frequency 
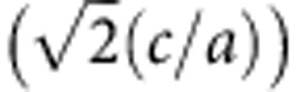
 of the high-order modes, there are two singly degenerate transverse electromagnetic (TEM) modes (spin-up forward and spin-down backward modes), whose longitudinal field components are zero (*E*_*x*_=*H*_*x*_=0). The insets of Fig. 1c illustrate their eigenfield. In addition, the high-order mode is a non-TEM mode (*E*_*x*_≠0, *H*_*x*_≠0) and is a doubly degenerate (spin-up and spin-down) band due to the mirror symmetry about the *yz* plane. The PECs (*ɛ*=−*∞*, *μ*=1) and PMCs (*ɛ*=1, *μ*=−*∞*) of the square waveguide satisfy the *ɛ*(*z*)=*μ*(−*z*) symmetry because they form a mirror pair about the *xy* plane. Once the spin-up forward mode is excited, the EM wave cannot be reflected in the absence of the backward spin-up mode, as long as the *ɛ*(*z*)=*μ*(−*z*) symmetry is preserved (for the symmetry-broken case, see Supplementary Note 1). To demonstrate its robust transport property, we use COMSOL to simulate a deformed waveguide in Fig. 1d, where the PEC–PMC boundaries are shown in grey/yellow. A plane wave travels into the waveguide and excites the spin-up TEM mode propagating along the +*x* direction. Note that a spinful source (in-phase/out-out-phase electric and magnetic currents) is not required to excite the waveguide mode. As the spin-up forward mode is the only propagating mode allowed in the forward direction, it can be excited as long as the exciting field has a non-zero projection on the spin-up mode. Wave propagation in this waveguide is protected by the *ɛ*(*z*)=*μ*(−*z*) symmetry. The waveguide is first bent in the +*y* direction through an S-shaped bend and then squeezed into a narrow square waveguide with a side length of 
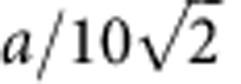
. After two 90° bends, it finally connects to a star-shaped waveguide. None of these deformations break the *ɛ*(*z*)=*μ*(−*z*) symmetry; therefore, the spin-up and spin-down states are still decoupled. In addition, no backscattering occurs in this waveguide, as can be seen from the *E*_*z*_ field pattern in Fig. 1e.

**Figure 2:**
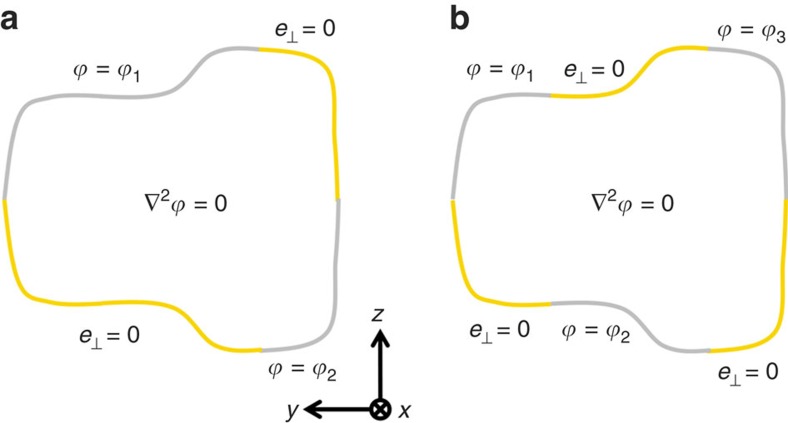
Number of TEM modes in the waveguide with PEC and PMC boundaries. Schematic of a waveguide having an arbitrary cross-section and satisfying the *ɛ*(*z*)=*μ*(−*z*) symmetry (**a**) with two PECs and two PMCs. When TEM modes are considered, Maxwell equations reduce to a Poisson's equation ∇^2^*ϕ*=0 with four boundary conditions. The two PMCs require the vertical component of electric field to be zero (*E*_⊥_=0), while the two PECs require the whole boundary has an equal potential *ϕ*_1_ or *ϕ*_2_. By applying the uniqueness theorem, one finds that this configuration has one spin-filtered TEM mode in each propagating direction. (**b**) A waveguide with three PECs and three PMCs. This configuration has two TEM modes in each direction. Spin-filtered feature is not guaranteed in this case.

It is important to note that the spin-filtered feature of our PEC–PMC waveguide does not depend on the shape of the PEC or PMC. In fact, it is determined by the number of PEC–PMC pairs in the boundaries and the *ɛ*(*z*)=*μ*(−*z*) symmetry. An arbitrarily shaped waveguide bounded by two PECs and two PMCs with its geometry satisfying *ɛ*(*z*)=*μ*(−*z*) symmetry is spin-filtered. Figure 2a depicts the general configuration of a waveguide consisting of two PECs and two PMCs. It can be proved that this waveguide having an arbitrary cross-section is spin-filtered (see Supplementary Note 2). It can further be shown (see Supplementary Note 2) that a waveguide satisfying *ɛ*(*z*)=*μ*(−*z*) symmetry and bounded by *N* pairs of PECs and PMCs will have (*N*−1) TEM modes for each propagation direction. The spin-filter feature is guaranteed for the case of *N*=2.

### Spin-filtered edge mode in simple structures

**Figure 3:**
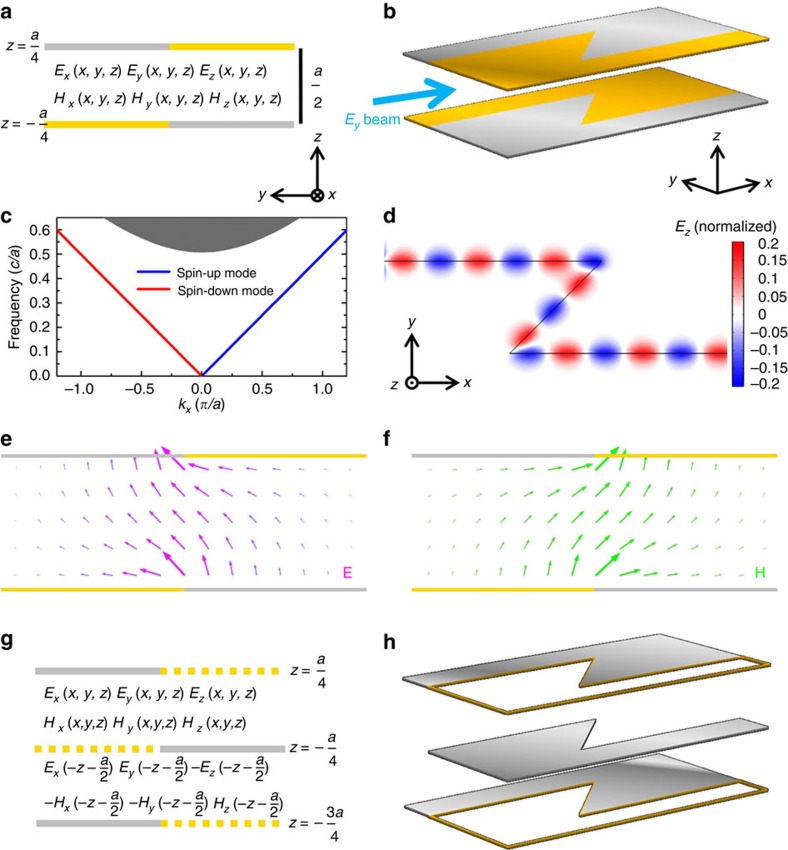
The spin-filtered edge mode between two parallel plate waveguides. (**a**) Cross-sectional view of an edge between two parallel plate waveguides, each of which consists of a PEC plate and a PMC plate. (**b**) Oblique view of a zigzag edge where *E*_*y*_-polarized EM wave is incident from the left. (**c**) Dispersion of the straight edge shown in **a**. The grey area represents the projected bulk modes in a single PEC–PMC parallel plate waveguide. Below its cutoff frequency of 0.5 (*c*/*a*), there exists only one spin-up forward edge mode (blue line) and one spin-down backward edge mode (red line). (**d**) *E*_*z*_ field pattern of the zigzag edge shown in **b** normalized by the incident field amplitude. The EM wave travels around two 135° corners without being reflected as the edge is spin-filtered. (**e**,**f**) Eigen electric field (magenta arrows) and magnetic field (green arrows) of the spin-up forward edge mode. To circumvent the complexity of using a PMC, we propose the periodic PEC configuration shown in **g**. The yellow dashed lines on *z*=−3*a*/4 and *z*=*a*/4 represent periodic boundaries. The periodic system in **g** has a field solution that corresponds to the solution of the system in **a**. Suppose the field solution of **a** consists of *E*_*x*_(*x*, *y*, *z*), *E*_*y*_(*x*, *y*, *z*), *E*_*z*_(*x*, *y*, *z*), *H*_*x*_(*x*, *y*, *z*), *H*_*y*_(*x*, *y*, *z*) and *H*_*z*_(*x*, *y*, *z*), which are non-zero in the domain *z*∈[−*a*/4, *a*/4]. The corresponding field solution of the periodic system is as illustrated in **g**. The spin-polarized robust transport in **d** can thus be realized for a broad range of frequencies by the periodic PEC plates shown in **h**.

Once we know how to achieve the spin-filter feature, we can use simple structures to manipulate EM wave in a scattering-free manner. Figure 3a shows the cross-section of another waveguide consisting of four parallel plates. The *ɛ*(*z*)=*μ*(−*z*) symmetry is preserved since the two PECs and the two PMCs form a pair of mirror images. The four parallel plates extend semi-infinitely in the *y* direction; therefore, the PECs (PMCs) in the upper half and the PMCs (PECs) in the lower half will meet at *y*→±∞. This waveguide thus supports two counterpropagating TEM modes whose pseudospins are locked to the wavevectors. Figure 3c plots its dispersion relation. The blue and red lines indicate the spin-up forward and spin-down backward TEM modes. Both TEM modes have a linear dispersion *ω*=2*πf*=*c*|*k*_*x*_|, where *c* is the speed of light. Figure 3e,f shows the eigen electric and magnetic fields of the forward spin-up mode. The electric and magnetic fields form an antimirror reflection, which is consistent with the definition of spin-up in equation (2). The fields are localized near the edge between the PECs and the PMCs. This field localization behaviour can be understood in the following way. The structure in Fig. 3a can be seen as a boundary between two parallel plate waveguides. The left waveguide has a PEC plate on top and a PMC plate at the bottom, while the right waveguide has a PMC plate on top and a PEC plate at the bottom. Both waveguides have a thickness of *a*/2 and a cutoff frequency of 0.5 (*c*/*a*). Below the cutoff frequency, no propagating EM mode exists for a single parallel plate waveguide. However, when the two parallel plate waveguides are interfaced with each other, the structure must support two TEM modes. Hence, the eigenfields of TEM modes must be edge modes localized near the boundary since the EM wave cannot propagate into sub-cutoff parallel plate waveguide on either side. One can mold the flow of EM wave in a two-dimensional plane in a scattering-free manner using this edge mode. The oblique view of a zigzag edge is shown in Fig. 3b, where the top and bottom plates are spaced further apart to reveal the geometry of the bottom plate. Figure 3d shows the *E*_*z*_ field pattern in the central plane. EM wave with a frequency of 0.3 (*c*/*a*) is incident from the left and guided along the edge (black line). In addition, no reflection occurs even when the EM wave propagates through two sharp corners. In addition, the PEC and PMC plates in Fig. 3a need not extend semi-infinitely in the y direction since the EM wave decays evanescently away from the edge.

The aforementioned spin-polarized waveguide or edge waveguide built from PECs and PMCs can, in principle, have a broad working bandwidth ranging from quasistatic wave up to the cutoff frequency. However, PMCs are typically realized with artificial subwavelength resonant structures^33^, which have a restricted bandwidth (see Supplementary Note 3). Instead of PMCs, we propose an implementation using the periodic PEC structure shown in Fig. 3g. Here we mimic an effective PMC boundary by introducing additional translation and mirror symmetries (see below). The yellow dashed lines indicate the whereabouts of the PMC plates in Fig. 3a. The yellow dashed lines on *z*=−3*a*/4 and *z*=*a*/4 also mark the periodic boundaries if we take the domain *z*∈[−3*a*/4, *a*/4] as a unit cell. The system is mirror-symmetric about the planes of *z*=±(2*N*−1)*a*/4 (*N*=1,2,3...) as it is periodic in the z direction. The eigenmodes can hence be classified as even or odd relative to the mirror reflections. For the even mode, its electric field is even under mirror reflection about *z*=±*a*/4. At the same time, the electric field at the yellow dashed line of *z*=−*a*/4 satisfies the continuous boundary condition. Hence, the electric field component perpendicular to the surface must be zero, as if an effective PMC boundary is placed at the yellow dashed line. Likewise, the continuous boundary condition (yellow dashed line) at *z*=*a*/4 also serves as effective PMC for the even mode. It is straightforward that the eigenfield of the periodic PEC structure (Fig. 3g) in the domain of *z*∈[−*a*/4, *a*/4] should be identical to the one of PEC–PMC structure (Fig. 3a). By performing a mirror reflection about *z*=−*a*/4 on the eigenfields in *z*∈[−*a*/4, *a*/4], the fields in *z*∈[−3*a*/4,−*a*/4] are obtained. Therefore, the eigenmodes of Fig. 3a and the even modes of Fig. 3g have one-to-one correspondence. In other words, suppose the field solution of Fig. 3a consists of *E*_*x*_(*x*, *y*, *z*), *E*_*y*_(*x*, *y*, *z*), *E*_*z*_(*x*, *y*, *z*), *H*_*x*_(*x*, *y*, *z*), *H*_*y*_(*x*, *y*, *z*) and *H*_*z*_(*x*, *y*, *z*), which are non-zero in the domain *z*∈[−*a*/4, *a*/4], then the periodic system in Fig. 3g must have a corresponding solution, as illustrated in Fig. 3g. In addition, the pseudospin-filtered edge mode localized near the edge should also exist in the periodic PEC structure.

On the other hand, the electric field for the odd mode should be odd under reflection about *z*=−*a*/4 and should be continuous at the yellow dashed line on *z*=−*a*/4. Thus, the electric field there is required to be perpendicular to the surface, as if an effective PEC boundary is there. Therefore, solving the odd modes in Fig. 3g is equivalent to solving the eigenmodes of a parallel plate waveguide where both the upper plate and the lower plate are PEC. The odd mode should be *E*_*z*_-polarized plane wave modes propagating in the *xy* plane, which are not localized near the edge. These extra odd modes are decoupled from and orthogonal to the even edge modes because of their different symmetries. The odd modes should not affect the transport of the edge mode.

**Figure 4:**
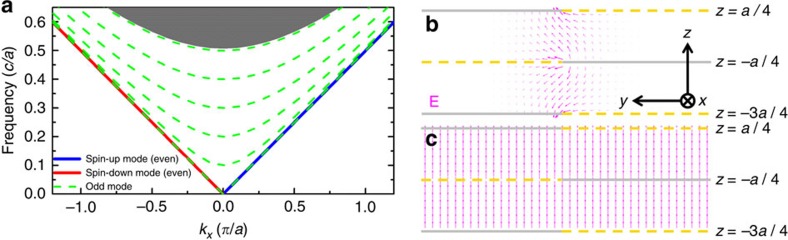
Dispersion and eigenfields of the periodic PEC structure. (**a**) Dispersion of the structure shown in Fig. 3g. Blue/red solid line denotes pseudospin-up/spin-down edge mode, which is even under reflection about *z*=−*a*/4. Green dashed lines denote *E*_*z*_-polarized bulk plane wave modes propagating in the *xy* plane, which is odd under mirror reflection. The grey area represents the projected bulk modes (even) in a single PEC parallel plate waveguide. (**b**) Eigen electric field of the spin-up mode at *k*_*x*_=0.5(*π*/*a*). (**c**) Eigen electric field of the lowest plane wave band at *k*_*x*_=0.5(*π*/*a*).

To demonstrate the field solution correspondence, we calculate the dispersion of periodic PEC edge waveguide in Fig. 3g. Results are shown in Fig. 4. Compared with the dispersion of the PEC–PMC edge waveguide (Fig. 3c), some additional bands of odd bulk modes emerge (green dashed lines in Fig. 4a), apart from the pseudospin-up/spin-down edge modes. Figure 4b plots the eigen electric field of the forward edge mode at *k*_*x*_=0.5(*π*/*a*). The eigenfields in the region of *z*∈[−*a*/4, *a*/4] is exactly the same as that in the PEC–PMC waveguide (Fig. 3e), while the fields in the domain *z*∈[−3*a*/4,−*a*/4] is its mirror image. This confirms our field solution correspondence. Figure 4c plots the electric field of the lowest additional band at *k*_*x*_=0.5(*π*/*a*). This is an *E*_*z*_-polarized plane wave bulk mode, which is odd under reflection. Note that periodic boundary condition is applied in the z direction (that is, *k*_*z*_=0) in the simulation. There should be other extra modes with *k*_*z*_≠0; however, they are decoupled from the *k*_*z*_=0 edge mode we concerned with as long as the translational symmetry along the *z* direction is preserved so that *k*_*z*_ is preserved.

Although the above discussion is about the eigen mode of a straight edge, the solution correspondence can be easily extended to the zigzag edge. The robust transport in Fig. 3d can be realized in a periodic PEC structure without using PMCs (Fig. 3h), and the edge mode would then have a truly broad working bandwidth. The translational symmetry along the *z* direction and the mirror symmetry ensure the correspondence of the field solution between the PEC–PMC structure and the periodic PEC configuration. We note that the *ɛ*(*z*)=*μ*(−*z*) symmetry in the original PEC–PMC structure ensures the decoupling between pseudospin-up and spin-down modes. Therefore, the robust transport of the edge mode in periodic PEC structure is protected by the translational symmetry, mirror symmetry and the hidden *ɛ*(*z*)=*μ*(−*z*) symmetry between PEC and effective PMC. Since these symmetries are preserved in the zigzag deformation of Fig. 3h, no reflection occurs at the two corners (see the result in Supplementary Fig. 8). We also note that the system in Fig. 3h employs PEC slabs with infinite periods. In actual implementation, a finite number of periods should be enough to realize spin-polarized robust transport (see Supplementary Note 5), although a small fraction of EM waves will leak from the topmost and bottommost periods. The small leakage, however, can be eliminated in a fan-shaped waveguide as shown below.

**Figure 5:**
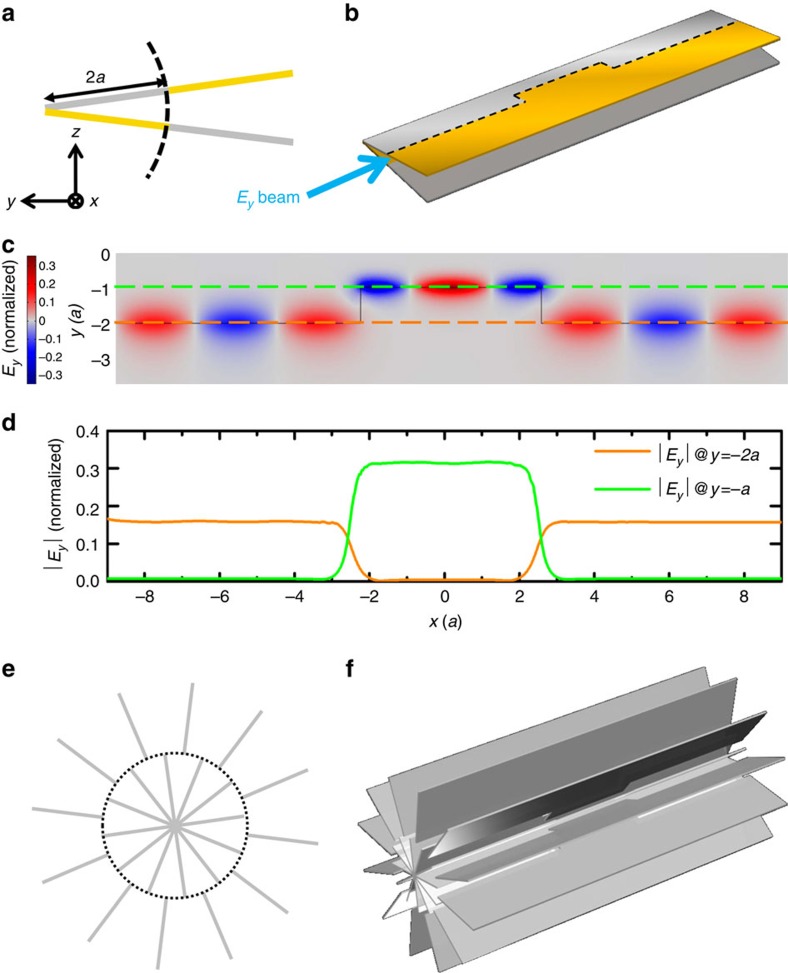
Field concentrator with broad bandwidth. (**a**) Cross-sectional view of an edge (black dashed line) between two fan-shaped plate waveguides. The distance between the apex and the edge is 2*a*. (**b**) Oblique view of a zigzag edge whose middle part is shifted closer (1*a*) to the apex. (**c**) *E*_*y*_ field pattern of the zigzag edge shown in **b** normalized by the incident field amplitude. When the EM wave is launched from the left, it travels through the shifted part without backscattering, which is verified by comparing the field amplitudes on the left- and right-hand sides. (**d**) Electric field amplitudes at the orange and green dashed lines in **c**, showing that the field amplitudes in the middle shifted edge region are doubled, as required by energy flux conservation. The edge between two fan-shaped plate waveguides can also be realized in the structure shown in **e**, which is periodic in the Azimuthal direction. (**f**) Oblique view of a field concentrator built from PEC slabs.

The robustness of our spin-polarized waveguide against symmetry-preserving deformation could lead to devices such as broadband field concentrators. EM field concentrators can be realized via tapered plasmonic waveguides^34^,^35^ or transformation optics^36^,^37^,^38^. Our spin-polarized waveguide provides an alternative way to focus an EM field for a broad range of frequencies and with a more compact structure. As an example, we consider the configuration shown schematically in Fig. 5a. It is similar to that in Fig. 3a; however, the top and bottom plates meet at an angle of 15° instead of being parallel. The edge between the PECs and the PMCs is 2*a* away from the apex. As the structure has *ɛ*(*z*)=*μ*(−*z*) symmetry and there are two PEC plates and two PMC plates, it is a spin-filtered channel. In Fig. 5b, we show the oblique view of a field concentrator that carries a defect region (5*a* in length) in the middle, where the edge is shifted closer to the apex (1*a* away from the apex). The EM wave guided along the edge will travel around the defect region without being reflected. The *E*_*y*_ field pattern shown in Fig. 5c (simulated by COMSOL) confirms the robust transport phenomenon, when an *E*_*y*_-polarized beam with a frequency of 0.22 (*c*/*a*) is incident from the left. Figure 5d plots the electric field amplitude |*E*_*y*_| at the two dashed lines marked in Fig. 5c. The electric field in the shifted region is enhanced by two times as that in the other region. This phenomenon can be understood in the following way. The defect region itself is another spin-filtered channel having two TEM modes. Its eigenfield pattern can be obtained by simply scaling down the eigenfield of the unshifted edge by two times. Thus, the eigenfield of the shifted edge should have a smaller beamwidth than the unshifted edge. In addition, there is no reflection at the boundary between the two different edges. To conserve the energy flow, the field amplitude in the defect region should be twice that of the unshifted edge. Similarly, one can increase the field concentration by decreasing the distance between the shifted edge and the apex. Figure 5e (cross-sectional view) and Fig. 5f (oblique view) show the realization of a broad bandwidth field concentrator using a PEC structure that is periodic in the Azimuthal direction. Its correspondence to the system in Fig. 5a can be proved in a similar way to the parallel plate waveguide (see Supplementary Note 4). The advantage of this fan-shaped waveguide is that the leakage of EM waves can be avoided since the edge (black dashed line in Fig. 5e) forms a closed loop. It should be emphasized that the edge transport in this periodic PEC fan-shaped waveguide is protected by *C*_12v_ symmetry and the hidden *ɛ*(*z*)=*μ*(−*z*) symmetry between PEC and effective PMC (see Supplementary Note 4).

### Experimental indication of a pseudospin-polarized waveguide

**Figure 6:**
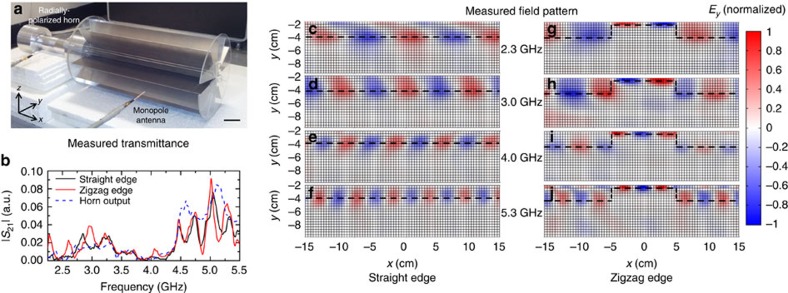
Experimental indication of a pseudospin-polarized waveguide. (**a**) The experimental set-up. Scale bar, 5 cm. EM wave was emitted from a radially polarized horn on the left of the sample. The electric field inside the sample was measured with a monopole antenna mounted on a stepmotor. (**b**) Measured transmittance of the sample with a straight edge (black line) or a zigzag edge (red line). The shifted region in the zigzag edge did not introduce obvious backscattering. Robust transport was also verified by the measured *E*_*y*_ field patterns. (**c**–**f**) Measured *E*_*y*_ fields in the sample with straight edges at 2.3, 3.0, 4.0 and 5.3 GHz. (**g**–**j**) Measured *E*_*y*_ fields in the sample with zigzag edge. Black dashed lines highlight the locations of edges. EM waves were always guided along the straight or zigzag edge. The field amplitudes on the left and right of the zigzag edge are almost the same, indicating robust transport. The fields in the shifted region were enhanced, compared with the unshifted region. The measured field patterns at each frequency are normalized by the maximal amplitude in the zigzag edge.

Figure 6 illustrates an experiment of our spin-polarized waveguide and field concentrator. In our microwave experiment, a periodic fan-shaped PEC waveguide was used to mimic the ideal PEC–PMC structure in Fig. 5a. Two samples were fabricated, where one had a straight edge and the other had a zigzag edge as in Fig. 5f. Both samples were built using 24 aluminium plates that were supported by two sheets of plexiglass. The length *a*=2 cm (see Fig. 5a) in our experiment. The experimental set-up and a photo of a sample are illustrated in Fig. 6a. The EM wave from a radially polarized horn impinged on the left of the sample (see Methods). The *E*_*y*_ field inside the sample was measured with a monopole antenna along the *y* direction. By stepping the antenna in increments of 4 mm × 4 mm in the *xy* plane, the field amplitude and phase were recorded. Figure 6b shows the measured transmittance of the sample with a straight edge (black line) or a zigzag edge (red line). The two spectra are essentially identical, implying that the defect region did not introduce back reflection and indicating pseudospin-polarized transport at frequencies ranging from 2.2 to 5.5 GHz. This also validates our approach of mimicking the PMC boundary for a broad-frequency range with a periodic structure. We note that the transmittances from 3.8 to 4.3 GHz were lower than other frequencies because of the lower emission power of the horn in this range. The blue dashed line in Fig. 6b plots the output spectra of the radial horn, which was measured without the sample. Figure 6c–f/g–j shows and compares the measured *E*_*y*_ field inside the sample with straight/zigzag edges at 2.3, 3.0, 4.0 and 5.3 GHz. The measured field patterns at each frequency are normalized by the maximal amplitude in the zigzag edge. The EM waves were always guided along the straight or zigzag edges (marked by black dashed lines). From Fig. 6g–j, the field amplitudes on the left and right of the zigzag edges are almost the same, indicating robust transport. We also found that the fields in the defect region were enhanced, compared with the fields in the unshifted region or in the straight edge, indicating the field concentrating effect. The field amplitudes in the middle shifted edge region are doubled, as required by energy flux conservation.

## Discussion

In summary, we have proposed a simple route to realize a pseudospin-polarized waveguide without using bulk materials. Numerical simulations and experimental results show its robust transport property and broad working bandwidth. On the basis of our spin-polarized waveguide, a broadband field concentrator was designed and realized experimentally. The realization of pseudospin transport simply by imposing boundary conditions has the obvious advantages of using less bulk material and being less lossy as the wave is guided in air. The idea may also be extended to the high-frequency regime, although the metallic loss limits the propagation distance (see Supplementary Note 6).

## Methods

### Experimental set-up

To excite the fully symmetric edge mode, we employ a radially polarized source whose electric field vector lies in the radial direction. Supplementary Fig. 14 gives the cross-sectional view of the radial horn. It consists of a circular PEC waveguide and a PEC rod lying at the centre of circle. Its fundamental mode is radially polarized and its electric field vectors point from the centred PEC rod to the outer circular waveguide. A monopole antenna was inserted into the waveguide to excite the fundamental mode. The tapered section on the right spans the radial beamwidth to accomodate the radius of the edge mode of the sample.

## Additional information

**How to cite this article:** Chen, W.-J. *et al.* Symmetry-protected transport in a pseudospin-polarized waveguide. *Nat. Commun.* 6:8183 doi: 10.1038/ncomms9183 (2015).

## Supplementary Material

Supplementary Information Supplementary Figures 1-14 and Supplementary Notes 1-6.

## References

[b1] Espinola R. L., Izuhara T., Tsai M.-C., Osgood R. M. & Dötsch H. Magneto-optical nonreciprocal phase shift in garnet/silicon-on-insulator waveguides. Opt. Lett. 9, 941–943 (2004) . 15143634 10.1364/ol.29.000941

[b2] Zaman T. R., Guo X. & Ram R. J. Faraday rotation in an InP waveguide. Appl. Phys. Lett. 90, 023514 (2007) .

[b3] Bi L. *et al.* On-chip optical isolation in monolithically integrated non-reciprocal optical resonators. Nat. Photon 5, 758–762 (2011) .

[b4] Gallo K., Assanto G., Parameswaran K. R. & Fejer M. M. All-optical diode in a periodically poled lithium niobate waveguide. Appl. Phys. Lett. 79, 314–316 (2001) .

[b5] Soljačić M., Luo C., Joannopoulos J. D. & Fan S. Nonlinear photonic microdevices for optical integration. Opt. Lett. 28, 637–639 (2003) . 12703925 10.1364/ol.28.000637

[b6] Fan L. *et al.* An all-silicon passive optical diode. Science 335, 447–450 (2012) . 22194410 10.1126/science.1214383 PMC5563475

[b7] Yu Z. & Fan S. Complete optical isolation created by indirect interband photonic transitions. Nat. Photon 3, 91–94 (2009) .

[b8] Kang M. S., Butsch A. & Russell P. St J. Reconfigurable light-driven opto-acoustic isolators in photonic crystal fibre. Nat. Photon 5, 549–553 (2011) .

[b9] Lira H., Yu Z., Fan S. & Lipson M. Electrically driven nonreciprocity induced by interband photonic transition on a silicon chip. Phys. Rev. Lett. 109, 033901 (2012) . 22861851 10.1103/PhysRevLett.109.033901

[b10] Haldane F. D. M. & Raghu S. Possible realization of directional optical waveguides in photonic crystals with broken time-reversal symmetry. Phys. Rev. Lett. 100, 013904 (2008) . 18232766 10.1103/PhysRevLett.100.013904

[b11] Wang Z., Chong Y., Joannopoulos J. D. & Soljačić M. Observation of unidirectional backscattering-immune topological electromagnetic states. Nature 461, 772–776 (2009) . 19812669 10.1038/nature08293

[b12] Ao X., Lin Z. & Chan C. T. One-way edge mode in a magneto-optical honeycomb photonic crystal. Phys. Rev. B 80, 033105 (2009) .

[b13] Fang K., Yu Z. & Fan S. Realizing effective magnetic field for photons by controlling the phase of dynamic modulation. Nat. Photon 6, 782–787 (2012) .

[b14] Haldane F. D. M. Model for a quantum Hall effect without Landau levels: condensed-matter realization of the “parity anomaly”. Phys. Rev. Lett. 61, 2015–2018 (1988) . 10038961 10.1103/PhysRevLett.61.2015

[b15] Jungwirth T., Niu Q. & MacDonald A. H. Anomalous Hall effect in ferromagnetic semiconductors. Phys. Rev. Lett. 88, 207208 (2002) . 12005602 10.1103/PhysRevLett.88.207208

[b16] Kane C. L. & Mele E. J. Quantum spin Hall effect in graphene. Phys. Rev. Lett. 95, 226801 (2005) . 16384250 10.1103/PhysRevLett.95.226801

[b17] Abanin D. A., Lee P. A. & Levitov L. S. Spin-filtered edge states and quantum Hall effect in graphene. Phys. Rev. Lett. 96, 176803 (2006) . 16712323 10.1103/PhysRevLett.96.176803

[b18] Murakami S. Quantum spin Hall effect and enhanced magnetic response by spin-orbit coupling. Phys. Rev. Lett. 97, 236805 (2006) . 17280226 10.1103/PhysRevLett.97.236805

[b19] Moore J. E. The birth of topological insulators. Nature 464, 194–198 (2010) . 20220837 10.1038/nature08916

[b20] Xia Y. *et al.* Observation of a large-gap topological-insulator class with a single Dirac cone on the surface. Nat. Phys. 5, 398–402 (2009) .

[b21] König M. *et al.* Quantum spin Hall insulator state in HgTe quantum wells. Science 318, 766–770 (2007) . 17885096 10.1126/science.1148047

[b22] Novoselov K. S. *et al.* Room-temperature quantum Hall effect in graphene. Science 315, 1379 (2007) . 17303717 10.1126/science.1137201

[b23] Hafezi M., Demler E. A., Lukin M. D. & Taylor J. M. Robust optical delay lines with topological protection. Nat. Phys. 7, 907–912 (2011) .

[b24] Hafezi M., Mittal S., Fan J., Migdall A. & Taylor J. M. Imaging topological edge states in silicon photonics. Nat. Photon 7, 1001–1005 (2013) .

[b25] Liang G. Q. & Chong Y. D. Optical resonator analog of a two-dimensional topological insulator. Phys. Rev. Lett. 110, 203904 (2013) . 25167412 10.1103/PhysRevLett.110.203904

[b26] Khanikaev A. B. *et al.* Photonic topological insulators. Nat. Mater. 12, 233–239 (2012) . 23241532 10.1038/nmat3520

[b27] Chen W.-J. *et al.* Experimental realization of photonic topological insulator in a uniaxial metacrystal waveguide. Nat. Commun. 5, 5782 (2014) . 25517229 10.1038/ncomms6782

[b28] Lu L., Joannopoulos J. D. & Soljačić M. Topological photonics. Nat. Photon 8, 821–829 (2014) .

[b29] Shomroni I. *et al.* All-optical routing of single photons by a one-atom switch controlled by a single photon. Science 345, 903–906 (2014) . 25146283 10.1126/science.1254699

[b30] Mitsch R., Sayrin C., Albrecht B., Schneeweiss P. & Rauschenbeutel A. Quantum state-controlled directional spontaneous emission of photons into a nanophotonic waveguide. Nat. Commun. 5, 5713 (2014) . 25502565 10.1038/ncomms6713 PMC4284658

[b31] Silveirinha M. & Engheta N. Tunneling of electromagnetic energy through subwavelength channels and bends using ɛ-near-zero materials. Phys. Rev. Lett. 97, 157403 (2006) . 17155357 10.1103/PhysRevLett.97.157403

[b32] Edwards B., Alu A., Young M. E., Silveirinha M. & Engheta N. Experimental verification of epsilon-near-zero metamaterial coupling and energy squeezing using a microwave waveguide. Phys. Rev. Lett. 100, 033903 (2008) . 18232982 10.1103/PhysRevLett.100.033903

[b33] Sievenpiper D., Zhang L., Broas R. F. J., Alexópolous N. G. & Yablonovitch E. High-impedance electromagnetic surfaces with a forbidden frequency band. IEEE Trans. Microwave Theory Tech. 47, 2059–2074 (1999) .

[b34] Stockman M. I. Nanofocusing of optical energy in tapered plasmonic waveguides. Phys. Rev. Lett. 93, 137404 (2004) . 15524758 10.1103/PhysRevLett.93.137404

[b35] Verhagen E., Spasenović M., Polman A. & Kuipers L. Nanowire plasmon excitation by adiabatic mode transformation. Phys. Rev. Lett. 102, 203904 (2009) . 19519030 10.1103/PhysRevLett.102.203904

[b36] Rahm M. *et al.* Design of electromagnetic cloaks and concentrators using form-invariant coordinate transformations of Maxwell's equations. Photonic. Nanostruct. 6, 87–95 (2008) .

[b37] Leonhardt U. Optical conformal mapping. Science 312, 1777–1780 (2006) . 16728596 10.1126/science.1126493

[b38] Pendry J. B., Schurig D. & Smith D. R. Controlling electromagnetic fields. Science 312, 1780–1782 (2006) . 16728597 10.1126/science.1125907

