# Supplementary material for: Symmetry-protected transport in a pseudospin-polarized waveguide
Source: Nat Commun. 2015 Sep 23;6:8183. doi: 10.1038/ncomms9183 (PMC4598361; doi:10.1038/ncomms9183)
Supplement: Supplementary Information — Supplementary Figures 1-14 and Supplementary Notes 1-6. [file ncomms9183-s1.pdf]

Supplementary Figures

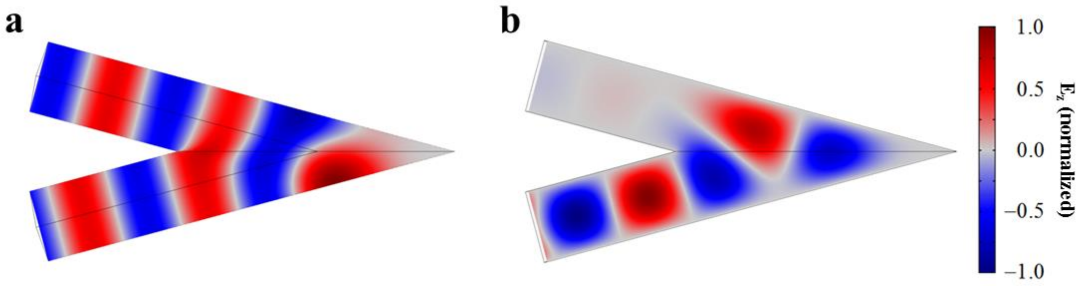

Supplementary Figure 1 | EM wave transport through a 150° bend. (a) Bend of our PEC-PMC waveguide. (b) Bend of the conventional PEC waveguide. Waves are incident from the lower left entrance of the waveguides. Note the absence of reflection in (a) and strong reflection in (b). The electric fields are normalized by the incident field.

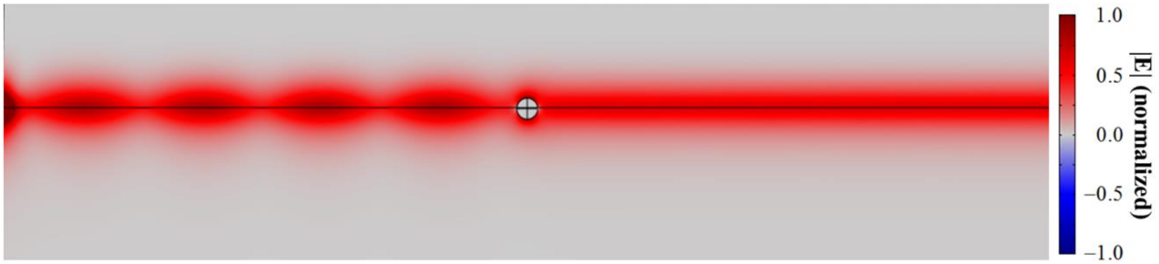

Supplementary Figure 2 | Backscattering introduced by a symmetry-breaking scatterer. A PEC sphere is put into the fan-shaped PEC-PMC waveguide. Electric field amplitude is plotted to see the interference fringe induced by reflection.

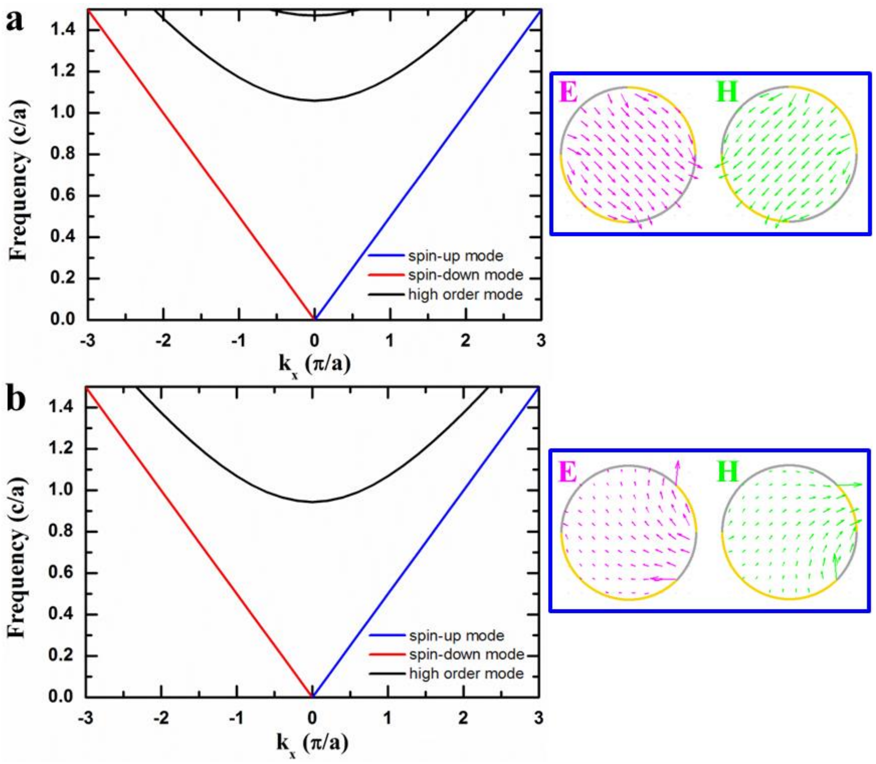

Supplementary Figure 3 | Dispersions and eigen fields of the circular waveguides with two PECs and two PMCs. In (a), each conductor subtends a 90° arc. In (b), the PEC (grey) and PMC (yellow) in the upper half space subtends 135° and 45° arcs, respectively. Right panels show the eigen electric (magenta arrows) and magnetic fields (green arrows) of the corresponding forward TEM modes.

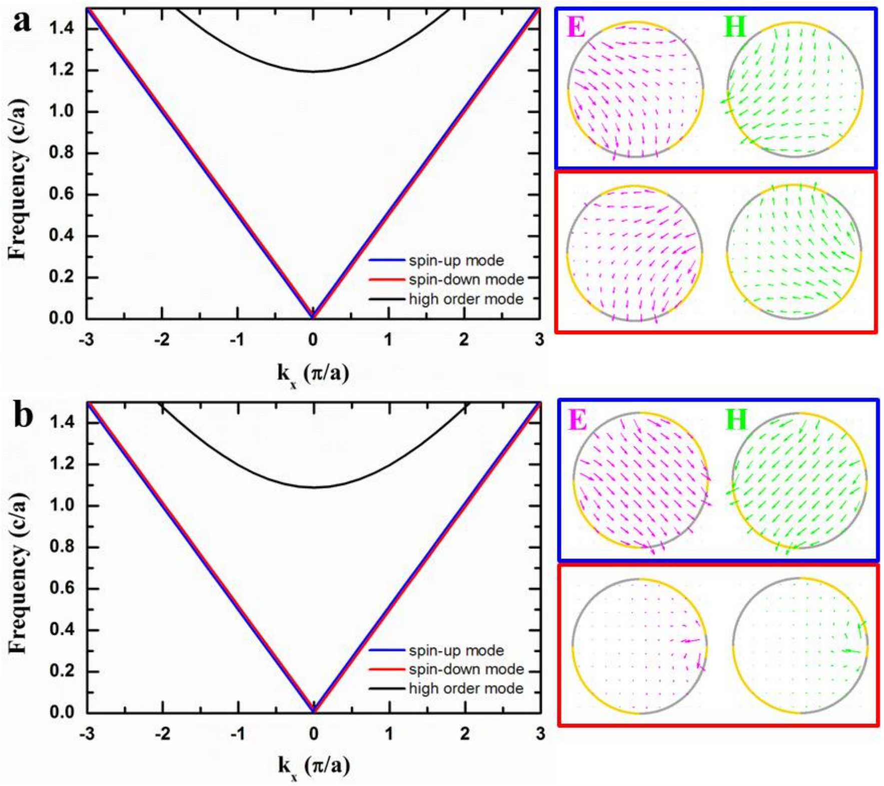

Supplementary Figure 4 | Dispersions and eigen fields of the circular waveguides with three PECs and three PMCs. In the right panels of (a) and (b), PECs and PMCs are highlighted by grey and yellow. In (a), each conductor subtends a 60° arc. In (b), the two PECs in the upper half space subtend 90° and 10° arcs, respectively. Right panels show the eigen electric (magenta arrows) and magnetic fields (green arrows) of the corresponding forward TEM modes. There are two TEM modes in each propagation direction.

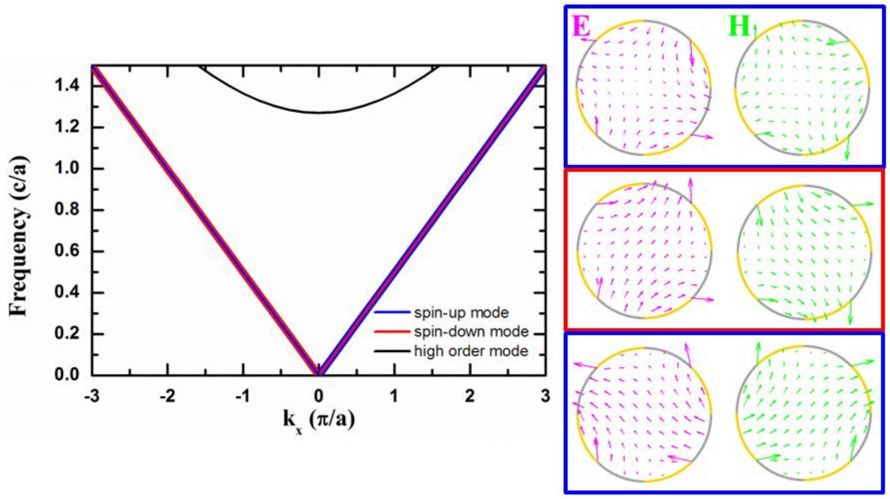

Supplementary Figure 5 | Dispersions and eigen fields of the circular waveguides with four PECs and four PMCs. In the right panels of (a) and (b), PECs and PMCs are highlighted by grey and yellow. Each conductor subtends a 45° arc. Right panels show the eigen electric (magenta arrows) and magnetic fields (green arrows) of the corresponding forward TEM modes. There are three TEM modes in each propagation direction.

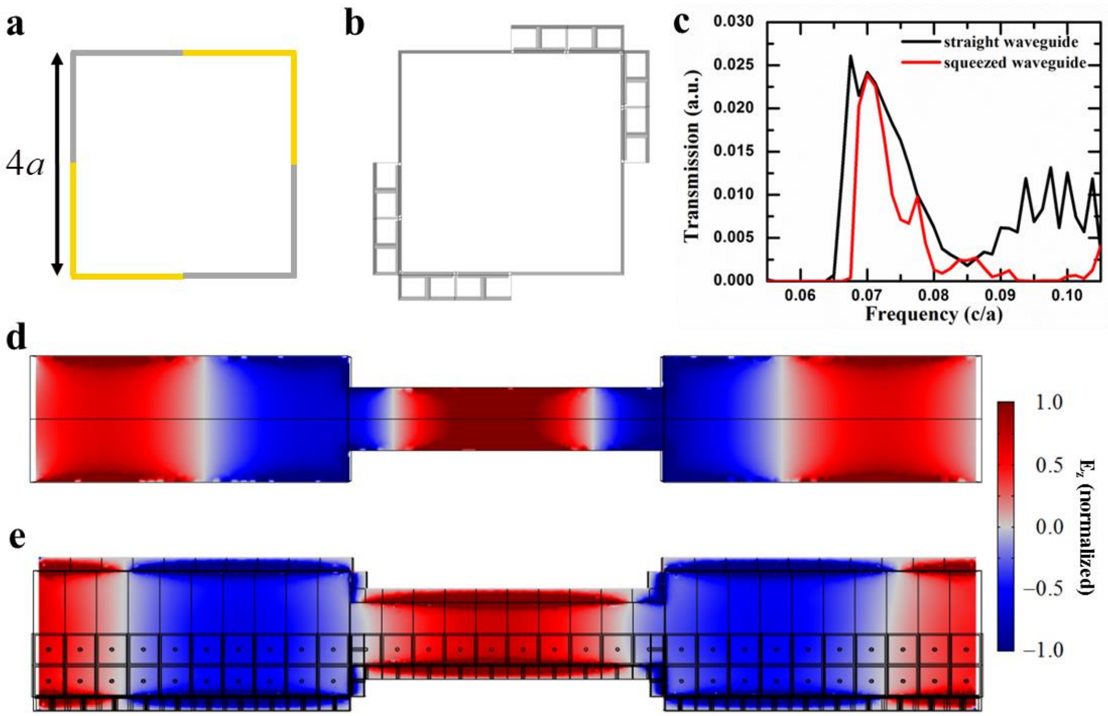

Supplementary Figure 6 | Spin-polarized waveguide with artificial magnetic conductors. (a) Cross sectional view of a square waveguide with “ideal” PMC boundaries marked by yellow color. (b) Cross sectional view of a square waveguide lined with artificial magnetic conductors implemented using high impedance surfaces (metallic mushroom arrays with resonance at 0.073 (c/a)). (c) Transmission spectra of the waveguide implemented by artificial magnetic conductors. (d) E_z field pattern in a square waveguide implemented by PMC, with a middle squeezed section. (e) E_z field pattern in a square waveguide implemented by artificial magnetic conductor.

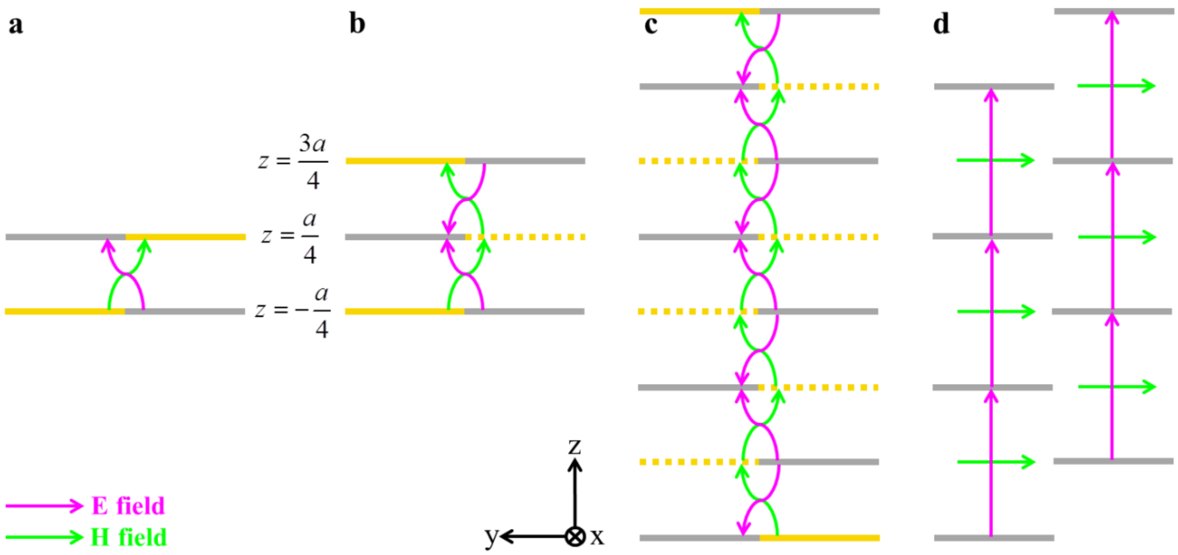

Supplementary Figure 7 | Field solution correspondence between PEC-PMC edge waveguide and periodic PEC edge waveguide. (a) Sketch diagram of the forward edge mode in the edge waveguide of Figure 3a, with the magenta (green) arrows indicating the direction of the electric (magnetic) field. (b) Stack another edge waveguide, which is flipped upside down, onto this edge waveguide. The eigen field in the new waveguide and the field in the original waveguide form a mirror reflection about $z = a/4$, and they are continuous at the common PMC boundary (yellow dashed line). Note that this PMC slab (yellow dashed line) can be removed without changing the eigenfield. This process can be repeated in both up and down directions, see (c). The edge mode should also exist in the periodic PEC structure of Figure 3g. Some extra "bulk" eigenmodes emerge below the original cutoff frequency with the removal of PMCs. One example is the E_z-polarized plane wave propagating along x direction, see (d). However, the edge mode in (c) and the plane wave bulk mode in (d) are decoupled from each other because of their different parities. The edge mode is even under mirror reflection about the planes $z = \pm(2N-1)a/4$ ($N = 1, 2, 3...$) while the plane wave mode is odd under reflection.

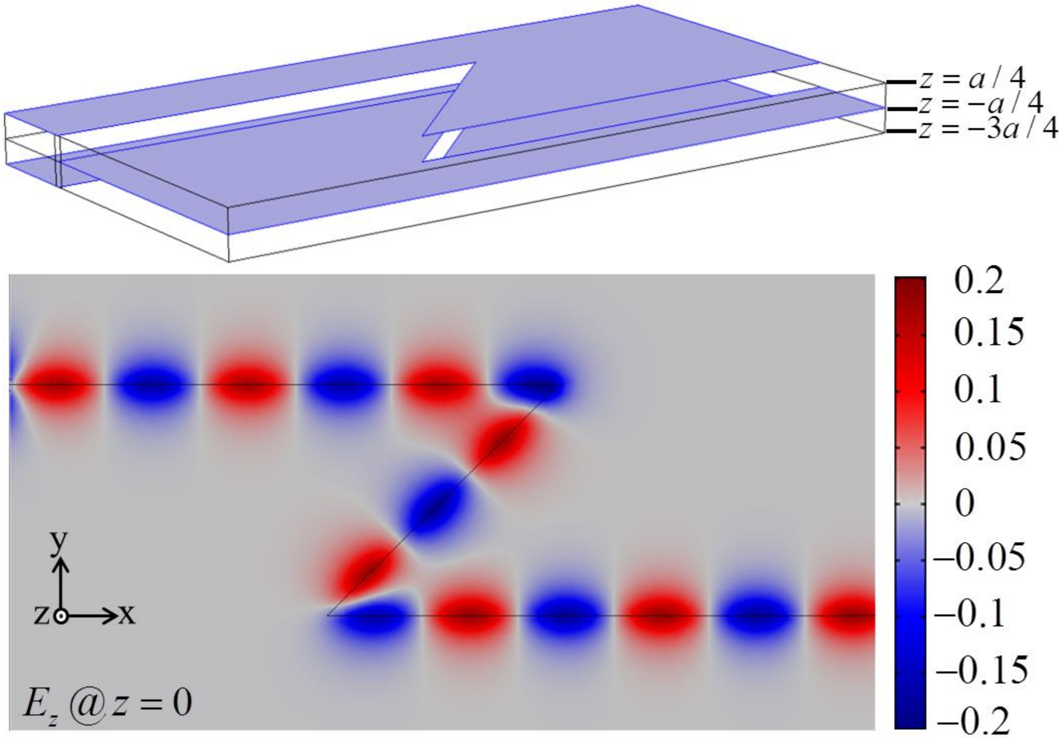

Supplementary Figure 8 | Robust transport at the zigzag edge of the periodic PEC. Upper panel shows the simulation setup for the zigzag edge in Figure 3h where periodic boundary condition is applied in the z direction. An E_y –polarized beam is incident from the left end of the zigzag edge. PEC boundaries are highlighted by blue. Lower panel plots the E_z field pattern at $z = 0$. It is identical to the field pattern in PEC-PMC zigzag edge shown in Figure 3d, which confirms the field solution correspondence.

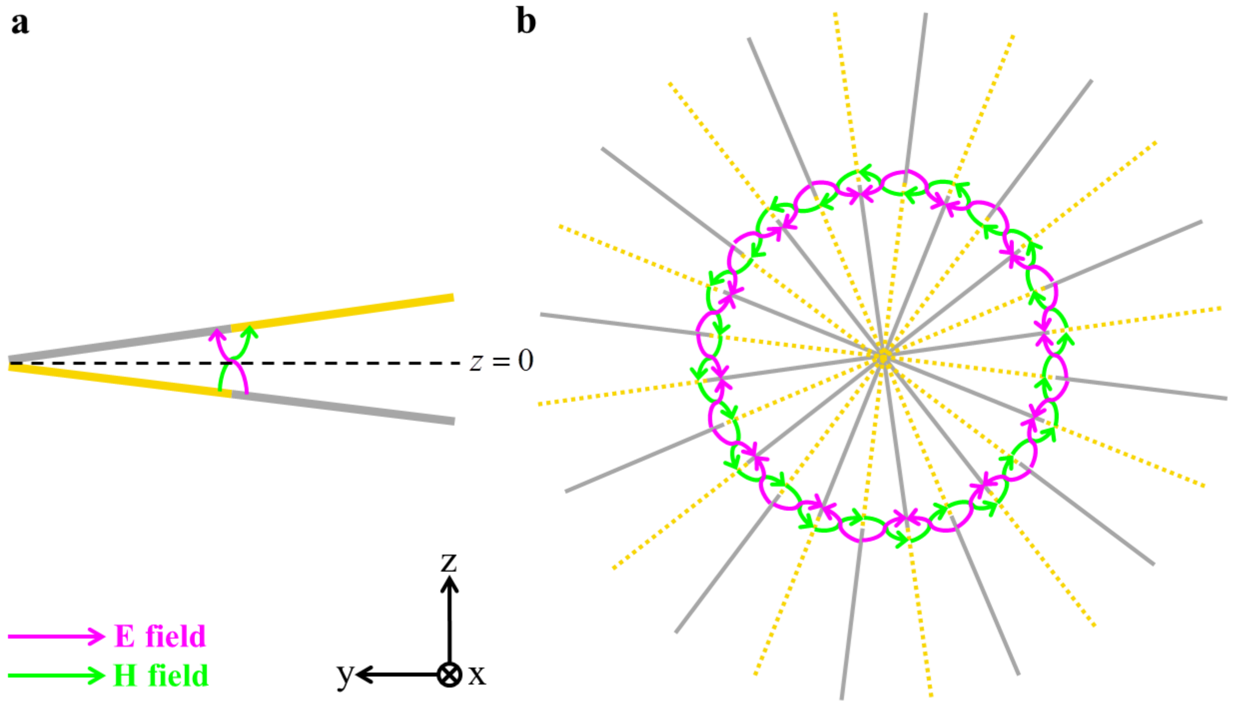

Supplementary Figure 9 | Field solution correspondence between PEC-PMC fan-shaped waveguide and PEC waveguide. (a) Sketch of the forward going edge mode in the PEC-PMC fan-shaped waveguide in Figure 5a, with magenta (green) arrows indicating the direction of the electric (magnetic) field. (b) Corresponding edge mode of the cyclic PEC fan-shaped waveguide in Figure 5e. Note that some extra eigenmodes (not confined to the edge) are introduced by the removal of PMC. But they are decoupled from the edge mode in (b) because they belong to different irreducible representations as long as the C_{12v} symmetry is preserved.

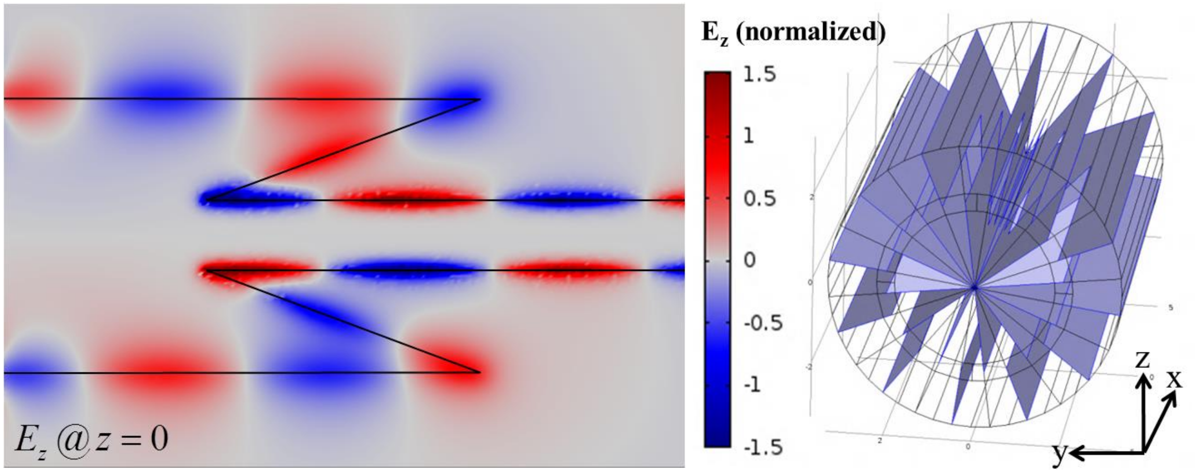

Supplementary Figure 10 | Robust transport at the zigzag edge of the PEC fan-shaped waveguide. Left panel plots the E_z field pattern at the plane $z = 0$. Right panel illustrates the PEC boundaries. A radially-polarized beam is incident from the left (–x direction).

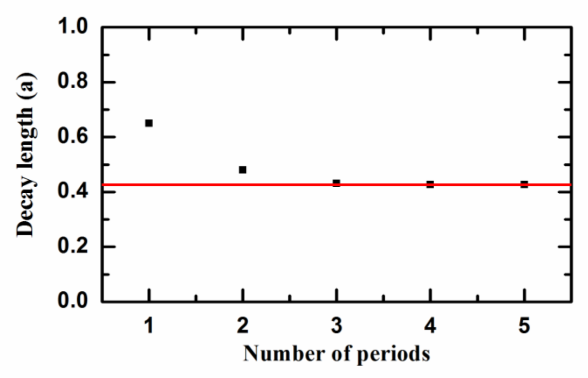

Supplementary Figure 11 | Decay length of the edge mode as a function of number of periods. The decay length is defined as the distance (in y-direction of Figure 3g) at which the magnitude of the electric field decays to 1/e of its value at the edge. Red line denotes the decay length for a periodic structure.

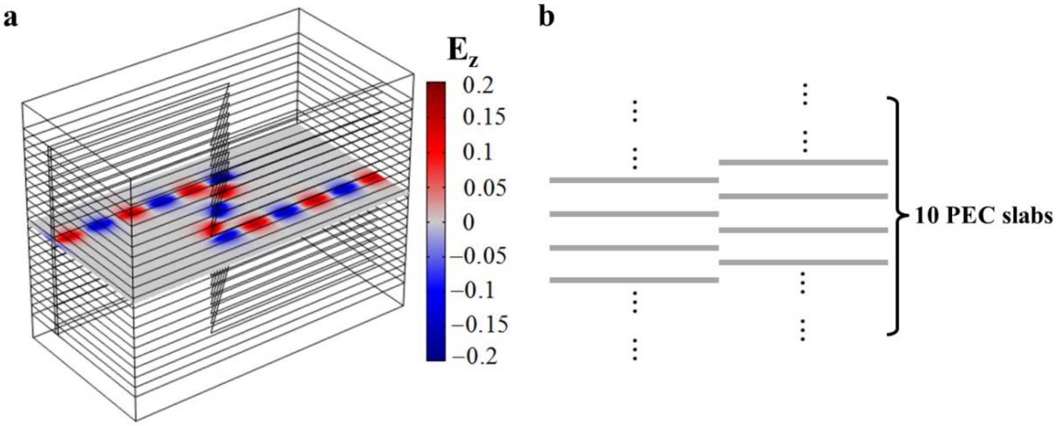

Supplementary Figure 12 | Realization of spin-polarized waveguide by periodic PEC slabs. (a) E_z field pattern at the middle plane of a zigzag edge waveguide. (b) Schematic view of the PEC slabs.

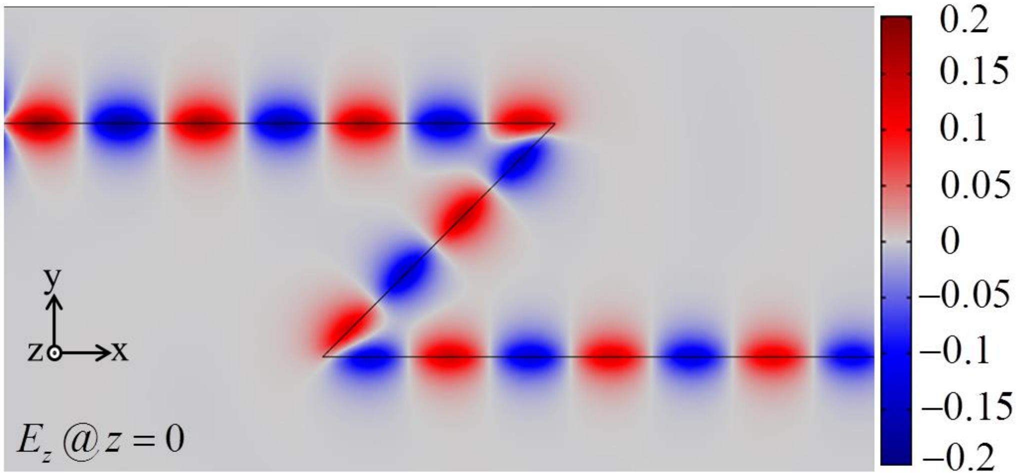

Supplementary Figure 13 | Robust transport in the high frequency regime. The period along z-direction is $a = 465\text{nm}$. The thickness of the gold slab is $0.1a = 46.5\text{nm}$. An E_y -polarized beam with frequency of $0.3\,(c/a)$ (wavelength of $1.55\mu\text{m}$) is incident from the left. The dielectric constant of gold $\varepsilon = -131.95 + 12.65i$ corresponds to the wavelength of $1.55\mu\text{m}$.

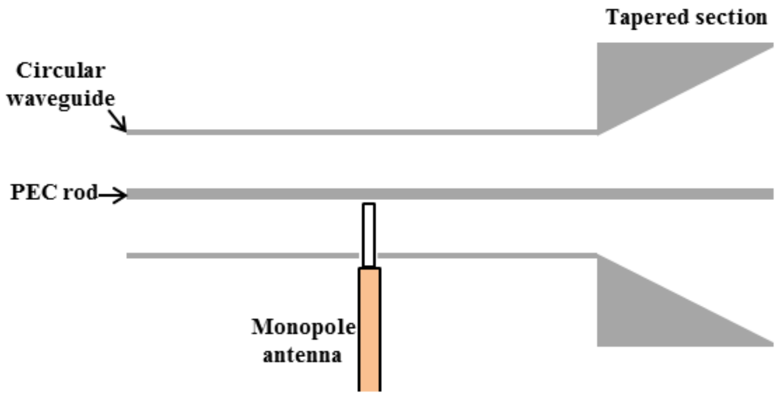

Supplementary Figure 14 | Cross-sectional view of the radially-polarized horn.

Supplementary Note 1

Symmetry protected transport in PEC-PMC waveguide

In order to clarify the robustness of the PEC-PMC waveguide in Figure 1b, we compare the wave propagations through a sharp bend in our PEC-PMC waveguide and in a conventional PEC waveguide. Supplementary Figure 1a simulates a 150° bend of the PEC-PMC waveguide with a configuration shown in Figure 1b in the text. EM wave is incident from the lower left entrance and then passes through the sharp bend without reflection. As a control calculation, we consider the case in a conventional PEC waveguide, which also has square cross section but all the four walls are bounded by PEC. Simulated electric field pattern is shown in Supplementary Figure 1b. The transmission through the bend is very weak and most of the EM waves are reflected.

In the cases discussed in the main text, deformations that preserve the $\varepsilon(z)=\mu(-z)$ symmetry will not introduce backscattering in spin-filtered waveguide. But when the deformation or scatterer breaks this symmetry, the two pseudospin states will couple to each other and backscattering occurs. Supplementary Figure 2 simulates a PEC sphere in the fan-shaped waveguide of Figure 5a. EM wave is incident from the left. The PEC sphere here is a symmetry-breaking scatterer, since its permittivity ($\varepsilon=-\infty$) and permeability ($\mu=1$) do not form a mirror reflection. Thus the spin-up forward mode can couple to the spin-down backward mode near the sphere and lead to reflection. This is verified in our simulation by the interference fringe on the left of the sphere.

Supplementary Note 2

Number of TEM modes in the waveguide with PEC and PMC boundaries

In the main text, we claimed that the spin-filtered feature is determined by the number of PEC/PMC boundaries and the $\varepsilon(z)=\mu(-z)$ symmetry. This claim is elaborated here. Figure 2a depicts a general configuration of a waveguide consisting of two PECs and two PMCs. Its geometry respects the $\varepsilon(z)=\mu(-z)$ symmetry, as the PEC (PMC) in upper half space and the PMC (PEC) in lower half form a pair of mirror images. The domain inside the waveguide is filled with air. We focus ourselves on the TEM modes, whose eigen fields can be written as

$$\mathbf{E}(x,y,z)=\begin{pmatrix}0 & e_y(y,z)e^{ik_x x} & e_z(y,z)e^{ik_x x}\end{pmatrix}^T,$$

$$\mathbf{H}(x,y,z)=\begin{pmatrix}0 & h_y(y,z)e^{ik_x x} & h_z(y,z)e^{ik_x x}\end{pmatrix}^T.$$

Plugging into Maxwell equations, one obtains a Poisson's equation $\nabla^2\varphi=0$, where $\mathbf{e}=(e_y,e_z)=-\nabla\varphi$ and $\mathbf{h}=(h_y,h_z)=(\mp e_z,\pm e_y)$ (the plus or minus sign depends on propagation direction). Then the TEM mode problem is equivalent to an electrostatics problem for $\mathbf{e}$ in a 2D space bounded by PEC and PMC, where we can determine the number of TEM waveguide mode by applying the uniqueness theorem for Poisson's equation. The uniqueness theorem states that the solution $\mathbf{e}$ is unique when all boundaries satisfy certain types of conditions. These conditions include (i) the electric field perpendicular to the boundary is well defined and (ii) the electric potential φ at the boundary is well defined. The two PMC boundaries in Figure 2a satisfy condition (i) obviously, as the electric fields perpendicular to the surface are zero ($e_\perp=0$). The two PEC boundaries will satisfy condition (ii) once their electric potentials φ_1 and φ_2 are given. Therefore the electric field $\mathbf{e}$ is unique once the potential difference $(\varphi_2-\varphi_1)$ is fixed. In addition, for different values of $(\varphi_2-\varphi_1)$, one can just multiply $\mathbf{e}$ by a constant factor. In this sense, in the electrostatic system exists only one eigen (linearly independent) solution for $\mathbf{e}$. Combining with $\mathbf{h}=(h_y,h_z)=(\mp e_z,\pm e_y)$, the waveguide shown in Figure 2a has only one TEM mode for each propagation direction. Because the waveguide has the $\varepsilon(z)=\mu(-z)$ symmetry, its Maxwell equations reduce to two equations for two pseudospins. Hence the forward TEM mode (propagating along $+x$ direction) must be spin-up polarized or spin-down polarized, i.e., its E_z and H_z components must be in-phase or out-of-phase. If the forward TEM mode is spin-up, then the backward mode must be spin-down by applying time reversal, and vice versa. Therefore we arrive at the conclusion that the waveguide shown in Figure 2a must be spin-filtered as long as the symmetry is preserved and the numbers of PEC are 2, independent of the cross-section shape.

Next we consider a waveguide with three PECs and three PMCs as depicted in Figure 2b. According to the uniqueness theorem, the electric field $\mathbf{e}$ is unique once the potential differences $(\varphi_2-\varphi_1)$ and $(\varphi_3-\varphi_1)$ are fixed. As there are two degrees of freedom in parameters, this linear system has two eigen modes, which can be proved in the following way. Suppose that the electric field solution is $\mathbf{e}_\mathrm{A}$ when $(\varphi_2-\varphi_1)=1$ and $(\varphi_3-\varphi_1)=0$, and the electric field solution is $\mathbf{e}_\mathrm{B}$ when $(\varphi_2-\varphi_1)=0$ and $(\varphi_3-\varphi_1)=1$. Then the field solution for other potential values must

be a linear combination of $\mathbf{e}_\text{A}$ and $\mathbf{e}_\text{B}$, that is to say, $\mathbf{e} = V_\text{A}\mathbf{e}_\text{A} + V_\text{B}\mathbf{e}_\text{B}$ when $(\varphi_2 - \varphi_1) = V_\text{A}$ and $(\varphi_3 - \varphi_1) = V_\text{B}$. Hence the waveguide in Figure 2b has two TEM modes for each propagation direction. However, the spin-filter feature cannot be guaranteed in this case, because the two TEM modes for forward (backward) direction can belong to different spins.

We have proved that a waveguide with two PECs and two PMCs has only one TEM mode for each direction and that its spin-filtered feature does not rely on the particular shape of PEC or PMC. Here we give some examples in the circular waveguide with PEC and PMC boundaries. The right panel of Supplementary Figure 3a shows a circular waveguide with two PECs (gray) and two PMCs (yellow), each conductor subtends a 90° arc. The radius of the circle is $0.25a$. The left panel of Supplementary Figure 3a calculates its dispersion. As expected, there exists a forward spin-up TEM mode (blue line) and a backward spin-down TEM mode (red line) below the cutoff frequency of high order waveguide mode (black line). The eigen electric (magenta arrows) and magnetic (green arrows) fields of its forward spin-up mode are illustrated in the right panel. In order to verify the influence of waveguide shape, we simulate another circular waveguide with two PECs and two PMCs (see the right panel of Supplementary Figure 3b), where the PEC in the upper half and the PMC in the lower half are changed to subtend 135° arc. The left panel of Supplementary Figure 3b demonstrates its spin-filtered feature. The dispersions of spin-up and spin-down modes keep unchanged on the line of $\omega = c|k_x|$, which is a characteristic of TEM mode, although their eigen fields change. In addition, the dispersion of high order mode and its cutoff frequency change with the waveguide shape.

Next we consider the circular waveguide with three PECs and three PMCs, each of which subtends a 60° arc (see Supplementary Figure 4a). It is seen from the left panel that the number of TEM modes for each direction is 2, which is consistent with our prediction. There exist one spin-up and one spin-down modes for both propagation directions. Thus the transport in this waveguide is not robust against deformation for the absence of spin-filtered feature. Note that the two spin TEM modes are doubly degenerate and are split a little in the figure for better illustration. The right panel of Supplementary Figure 4a shows the corresponding eigen fields for forward direction. Supplementary Figure 4b calculates another waveguide with changed geometry, where the two PECs in upper half (also the two PMCs in lower half) subtends a 90°

arc and a 10° arc. This change does not affect the number of TEM modes, see the dispersion in Supplementary Figure 4b.

It is straightforward to extend the argument to the waveguide with N alternate pairs of PECs and PMCs, which has $(N-1)$ degenerate TEM modes for each direction. Supplementary Figure 5 gives an example with $N=4$.

Supplementary Note 3

Spin-polarized waveguide with artificial magnetic conductors

Here we give an example of spin-polarized waveguide implemented by artificial magnetic conductor, which is implemented using the commonly-employed "high impedance surfaces". Supplementary Figure 6a gives the geometry of a square waveguide with ideal PMC, defined as a material with $\{\varepsilon=1,\ \mu=-\infty\}$. According to our previous discussion, this waveguide is robust against deformation if the $\varepsilon(z)=\mu(-z)$ symmetry is preserved. For instance, the middle part of the waveguide is squeezed into a narrower waveguide with a width of $2a$, see Supplementary Figure 6d. When a plane wave is incident from left, the EM wave can tunnel through the squeezed waveguide with total transmission. Next we consider the case with artificial magnetic conductor. The PMC boundaries (yellow lines) in Supplementary Figure 6a are then replaced by metallic mushroom arrays, see Supplementary Figure 6b. The mushroom structure has a square lattice and the lattice constant is a. Its resonant frequency, where this artificial structure behaves as a high impedance surface, is tuned to $0.073\,(c/a)$ by varying the dimension of mushroom. Supplementary Figure 6e plots the E_z field pattern found using COMSOL in the square waveguide implemented by artificial magnetic conductor. As we expected, EM wave tunnels through the narrow channel with high transmission. Corresponding transmission spectrum calculated using COMSOL is plotted by red line in Supplementary Figure 6c. For comparison, black line plots the transmission spectrum of a straight waveguide (without squeezed section) also implemented by mushroom array. Due to the resonant property of artificial magnetic conductor, the robust behavior can only be observed in a narrow frequency region near the resonant frequency.

Supplementary Note 4

The correspondence of the field solutions between the PEC-PMC structure and the periodic PEC structure

In this section, we elaborate the field solution correspondence between PEC-PMC structure and periodic PEC structure in detail. First, we take the PEC-PMC edge waveguide in Figure 3a as an example. As mentioned in the main text, the structure possesses two edge modes below the frequency of 0.5 (c/a). The EM fields of both modes are localized near the edge, and the electric/magnetic fields connect the two PEC/PMC slabs. The eigen fields of the forward (+x) edge mode is schematically plotted in Supplementary Figure 7a, with magenta (green) arrow representing the direction of the electric (magnetic) field. We can stack another edge waveguide, which is flipped upside down, onto the original edge waveguide. This new edge waveguide also possesses two edge modes, one going forward and one going backward, and the field pattern of its forward going eigen mode should be the mirror image of the original forward edge mode as shown in Supplementary Figure 7b. As a pseudo vector, magnetic field gains an additional sign flip under reflection. Thus both the magnetic fields in the region of $z \in [-a/4, a/4]$ and $z \in [a/4, 3a/4]$ point up, as illustrated in the Supplementary Figure 7b. One can prove that the EM fields in these two regions are continuous at their common PMC boundary ($z = -a/4$ and $y > 0$, yellow dashed line in Supplementary Figure 7b). Hence the eigen mode of the system with this PMC is also a solution of the system if we remove the PMC. In other words, the solution is the same whether the yellow dashed lines in Supplementary Figure 7b represents a PMC or air. We can further stack as many PEC-PMC waveguide together as we like and we remove the PMCs to form a periodic PEC structure, which is shown schematically in Supplementary Figure 7c. There also exists an edge mode, which is the solution for the system independent of whether the yellow dashed lines are PMCs or air.

Some extra “bulk” modes, which is not supported by the original PEC-PMC edge waveguide, emerge below the cutoff frequency after removing the PMC slabs. For example, an E_z-polarized plane wave propagating along +x direction (Supplementary Figure 7d), other than the edge mode in Supplementary Figure 7c, is supported by the periodic PEC structure. Nevertheless, these extra bulk modes are decoupled from the edge mode due to their different symmetries. The electric field of the edge mode is even under the reflection about the planes of $z = \pm(2N-1)a/4$ ($N = 1, 2, 3...$). On the other hand, the electric field of the plane wave bulk mode is odd under reflection. Therefore the extra modes should not affect the transport of the

edge mode. Meanwhile, the edge mode and the plane wave mode can be excited selectively by the source with even or odd parity. For instance, the edge mode can be selectively excited by an E_y –polarized beam (even under σ_z) while the plane wave mode can be excited by an E_z-polarized beam (odd under σ_z).

Although the discussion above is about the eigenmode of a straight edge, the solution correspondence can be easily extended to the zigzag edge. Supplementary Figure 8 simulates the robust transport at the zigzag edge of periodic PEC slabs. Blue color in the upper panel highlights the PEC boundaries. An E_y-polarized beam is incident from left to excite the even edge mode, which travels around two 135° corners without reflection. The E_z field pattern at $z=0$ is identical to that in Figure 3d and confirms our prediction. Remember that the robust transport of the edge mode in periodic PEC structure is protected by the translational symmetry, mirror symmetry and the hidden $\varepsilon(z)=\mu(-z)$ symmetry. Since these symmetries are preserved in the zigzag deformation of Supplementary Figure 8, no reflection occurs at the two corners.

The field solution correspondence between PEC-PMC fan-shaped waveguide and periodic PEC waveguide can be proved in a similar way. The eigen field of PEC-PMC fan-shaped waveguide is schematically shown in Supplementary Figure 9a, again with magenta (green) arrows representing the direction of the electric (magnetic) field. The corresponding edge mode in a PEC fan-shaped waveguide can be obtained by stacking 24 PEC-PMC waveguides together along the azimuthal direction and removing the PMCs, as shown in Supplementary Figure 9b. Extra "bulk" modes are introduced by removing the PMCs, but they are decoupled from the edge mode since the extra modes and the edge mode belong to different irreducible representations of C_{12v} symmetry group. The electric field edge mode is a fully symmetric (A_1) mode as both its rotation eigen values and mirror eigen values are 1. Thus a fully symmetric source is needed to excite the edge mode, e.g., the radially-polarized beam used in our experiment. The solution correspondence can also be extended to the zigzag edge. Simulated E_z field pattern in Supplementary Figure 10 demonstrates the robust transport at the zigzag edge of a PEC fan-shaped waveguide.

Similar to the periodic structure in Supplementary Figure 8, the robustness of the edge mode in PEC fan-shaped waveguide is protected by C_{12v} symmetry and the $\varepsilon(z)=\mu(-z)$ symmetry of its corresponding PEC-PMC structure. We can also comprehend this in another way.

Note that the yellow dashed lines in Supplementary Figure 9b also mark the mirror symmetry planes of this C_{12v} system. When we focus on the fully symmetric solution of the C_{12v} system, its electric field at the yellow lines should satisfy both the mirror symmetry (even under reflection) and the continuous boundary condition. These two conditions require the direction of the electric field to be parallel to these mirror planes (marked by yellow dashed lines), i.e., the electric field component perpendicular to these planes should be zero. In this way, the continuous boundary conditions lying on the mirror symmetry planes act as an effective PMC boundary. Therefore solving the fully symmetric solution (A_1 for E field) of the C_{12v} system in Supplementary Figure 9b is equivalent to solving the PEC-PMC problem in Supplementary Figure 9a. It is the C_{12v} symmetry that ensures this equivalence and the effective PMC boundary. Hence the edge transport is robust against arbitrary defect that preserves both C_{12v} symmetry and the hidden $\varepsilon(z)=\mu(-z)$ symmetry between the PEC and the effective PMC, the latter condition ensuring the pseudo spins' decoupling.

Supplementary Note 5

Realization in periodic PEC slabs with finite periods

According to the field solution correspondence, the edge mode of PEC-PMC waveguide in Figure 3a can be realized in a periodic PEC waveguide with infinite periods. For the PEC structure with a finite number of periods, the fields in the outermost layers should deviate from the infinitely periodic structure. It is a natural question to ask as to how many periods should be enough to realize the edge mode in the PEC-PMC configuration. We numerically calculate the decay length of the edge mode at the central plane ($z=0$) as a function of number of periods, plotted as the black squares in Supplementary Figure 11. The edge mode decay length is defined as the distance at which the magnitude of the electric field decays to 1/e of its value at the edge. For the extreme case of one single period (two PEC slabs), the central plane lies in the outermost layer. The eigen field of the edge mode is expected to deviate quite a bit from the periodic one. Its decay length is $0.65a$ compared with the ideal value of $0.426a$ (marked by the red line). The "one period" structure is a system with two PECs, and as such, it support one TEM edge mode according to our discussion in Supplementary Note 2. The eigen field of the TEM edge mode can be obtained by solving the Poisson's equation when we apply different electric potentials on the two PECs. And it is straightforward to see that most of the electric field localized near the edge.

As the number of periods increases, the central plane lies further and further away from the outermost layer and its eigen field returns to the ideal periodic one. As we can see from Supplementary Figure 11, when the central plane is 1.25a away from the outermost PEC slab (for the case of 3 periods), the decay length of the eigenfield is almost the same as the ideal one.

Here we give an example of realizing robust transport in periodic PEC structure like Figure 3h but with a finite number of periods. A periodic PEC slabs with 10 periods is simulated in Supplementary Figure 12a, where the PEC slabs have the same geometry as that in Figure 3h. Its cross sectional view is illustrated in Supplementary Figure 12b. The structure is illuminated by an E_y–polarized wave with frequency of $0.3\,(c/a)$ from left. The field pattern at the middle plane in Supplementary Figure 12a shows that the EM wave is guided through the zigzag edge without backscattering. All calculations are done using COMSOL.

Supplementary Note 6

Feasibility of the periodic PEC edge configuration in the high frequency regime

The dissipative loss of the metals should be taken into account in high frequency regimes and we expect that the loss will limit the propagation length of the edge mode. Supplementary Figure 13 simulates the zigzag edge of periodic gold slabs that is similar to Supplementary Figure 8. Here the period along z-direction is $a = 465\text{nm}$. The thickness of the gold slab is $0.1a = 46.5\text{nm}$. An E_y-polarized beam with frequency of $0.3\,(c/a)$ (wavelength of $1.55\mu\text{m}$) is incident from the left. The dielectric constant of gold $\varepsilon = -131.95 + 12.65i$ corresponds to the telecommunication wavelength of $1.55\mu\text{m}$. As can be seen from Supplementary Figure 13, the EM wave is still robustly guided along the zigzag edge although some decay exists along the propagation direction due to the metallic loss. The robust transport can still be sustained because the field of edge mode is mainly guided in air and therefore the mode propagation is not seriously compromised by the absorption of the metal.
